# Telehealth treatment of patients with major depressive disorder during the COVID-19 pandemic: Comparative safety, patient satisfaction, and effectiveness to prepandemic in-person treatment

**DOI:** 10.1192/j.eurpsy.2023.894

**Published:** 2023-07-19

**Authors:** M. Zimmerman

**Affiliations:** Brown University, Providence, United States

## Abstract

**Introduction:**

The COVID-19 pandemic impelled a transition from in-person to telehealth psychiatric treatment. The COVID-19 pandemic has been depressogenic. Major disruptions in lifestyle due to social isolation, job loss, financial strain, and deaths of neighbors, family and friends are potential contributors to the increased levels of depression due to the pandemic. As one of the core elements of psychotherapeutic approaches towards treating depression is behavioral activation and increased social contact the psychosocial limitations imposed by COVID-19 might make it more difficult to treat depression during the pandemic.

**Objectives:**

There are no studies of partial hospital telehealth treatment for major depressive disorder (MDD). In the present report from the Rhode Island Methods to Improve Diagnostic Assessment and Services (MIDAS) project, we compared the effectiveness of partial hospital care of patients with MDD treated virtually versus in-person.

**Methods:**

Outcome was compared in 294 patients who were treated virtually from May, 2020 to December, 2021 to 542 patients who were treated in the in-person partial program in the 2 years prior to the pandemic. Patients completed self-administered measures of patient satisfaction, symptoms, coping ability, functioning, and general well-being.

**Results:**

In both the in-person and telehealth groups patients with MDD were highly satisfied with treatment and reported a significant reduction in symptoms from admission to discharge. Both groups also reported a significant improvement in positive mental health, general well-being, coping ability, and functioning. A large effect size of treatment was found in both treatment groups. Contrary to our hypothesis, the small differences in outcome favored the telehealth-treated patients. The length of stay and the likelihood of staying in treatment until completion were significantly greater in the virtually treated patients.

**Conclusions:**

In an intensive acute care setting, delivering treatment to patients with MDD using a virtual, telehealth platform was as effective as treating patients in-person.

**Disclosure of Interest:**

None Declared

